# Measuring Cardiac Dyssynchrony with DENSE (Displacement Encoding with Stimulated Echoes)—A Systematic Review

**DOI:** 10.31083/j.rcm2409261

**Published:** 2023-09-18

**Authors:** Saara Sillanmäki, Hanna-Liina Vainio, Elias Ylä-Herttuala, Minna Husso, Marja Hedman

**Affiliations:** ^1^Institute of Medicine, University of Eastern Finland, 70210 Kuopio, Finland; ^2^Diagnostic Imaging Center, Kuopio University Hospital, 70029 Kuopio, Finland; ^3^A.I. Virtanen Institute, University of Eastern Finland, 70210 Kuopio, Finland; ^4^Heart Center, Kuopio University Hospital, 70029 Kuopio, Finland

**Keywords:** displacement encoding with stimulated echoes, DENSE, cardiovascular magnetic resonance imaging, CMR, MRI, dyssynchrony, systematic review

## Abstract

**Background:**

In this review, we introduce the displacement encoding with stimulated echoes (DENSE) method for measuring myocardial dyssynchrony using cardiovascular magnetic resonance (CMR) imaging. We provide an overview of research findings related to DENSE from the past two decades and discuss other techniques used for dyssynchrony evaluation. Additionally, the review discusses the potential uses of DENSE in clinical practice.

**Methods:**

A search was conducted to identify relevant articles published from January 2000 through January 2023 using the Scopus, Web of Science, PubMed and Cochrane databases. The following search term was used: (DENSE OR ‘displacement encoding with stimulated echoes’ OR CURE) AND (dyssynchrony* OR asynchron* OR synchron*) AND (MRI OR ‘magnetic resonance’ OR CMR).

**Results:**

After removing duplicates, researchers screened a total of 174 papers. Papers that were not related to the topic, reviews, general overview articles and case reports were excluded, leaving 35 articles for further analysis. Of these, 14 studies focused on cardiac dyssynchrony estimation with DENSE, while the remaining 21 studies served as background material. The studies used various methods for presenting synchronicity, such as circumferential uniformity ratio estimate (CURE), CURE-singular value decomposition (SVD), radial uniformity ratio estimate (RURE), longitudinal uniformity ratio estimate (LURE), time to onset of shortening (TOS) and dyssynchrony index (DI). Most of the dyssynchrony studies concentrated on human heart failure, but congenital heart diseases and obesity were also evaluated. The researchers found that DENSE demonstrated high reproducibility and was found useful for detecting cardiac resynchronisation therapy (CRT) responders, optimising CRT device settings and assessing right ventricle synchronicity. In addition, studies showed a correlation between cardiac fibrosis and mechanical dyssynchrony in humans, as well as a decrease in the synchrony of contraction in the left ventricle in obese mice.

**Conclusions:**

DENSE shows promise as a tool for quantifying myocardial function and dyssynchrony, with advantages over other cardiac dyssynchrony evaluation methods. However, there remain challenges related to DENSE due to the relatively time-consuming imaging and analysis process. Improvements in imaging and analysing technology, as well as possible artificial intelligence solutions, may help overcome these challenges and lead to more widespread clinical use of DENSE.

## 1. Introduction

Cardiovascular magnetic resonance (CMR) imaging plays an increasingly important role in routine cardiology clinical practice. It is a safe, radiation-free and non-invasive imaging modality used in the evaluation of cardiac structures and function. The first method for measuring intramyocardial motion, tagging, was introduced by Zerhouni *et al*. [1] over 30 years ago. Since the late 90s, several novel technologies have been introduced for CMR-based measurement of myocardial motion. In 1999, Aletras *et al*. [2] introduced displacement encoding with stimulated echoes (DENSE) as a tool for measuring left ventricular (LV) motion. The development of CMR imaging is an active field of research that has seen a rapid expansion of new and emerging techniques. For instance, there have been advancements in shortening scanning and processing times [3, 4], improving signal quality through different applications [5] and achieving excellent reproducibility [6, 7]. Also, automatic analyzing tools have been developed to reduce the need for manual delineation of anatomical structures [8, 9, 10]. Moreover, nowadays, myocardial contraction can be analyzed from both the left and right ventricles with CMR [11, 12]. In addition, advances in CMR technology have led to the introduction of three-dimensional (3D) methods for the measurement of myocardial motion, as reported in several studies [13, 14, 15, 16, 17].

There are a variety of methods available to measure LV dyssynchrony, including the measurement of QRS complex duration via electrocardiogram, as well as echocardiography and CMR-based measurements. Failure in the timing of myocardial contraction can lead to a disruption in cardiac output, which can in turn lead to heart failure (HF). By identifying cardiac dyssynchrony, healthcare professionals can take proactive steps to prevent the progression of HF. One of the most important applications of dyssynchrony measurement is in the selection of patients for cardiac resynchronization therapy (CRT) [18, 19]. While CRT can be effective for many patients, not all individuals respond positively to the treatment. Additionally, in certain cases, the installation of a CRT device can worsen the existing dyssynchrony instead of improving it [20]. Moreover, not only can DENSE provide important information on myocardial motion and deformation, but novel studies have shown that DENSE can also be used to measure cardiac volumes and myocardial mass [21]. Research on the DENSE technique is not limited only to human studies but also includes preclinical studies in animals and phantoms [22, 23, 24, 25, 26, 27]. For the abovementioned reasons, DENSE could also have value in developing and testing new drugs and other therapeutic interventions for HF [28, 29].

## 2. Measurement of Cardiac Dyssynchrony with DENSE

### 2.1 Data Acquisition

**Fig. 1.**
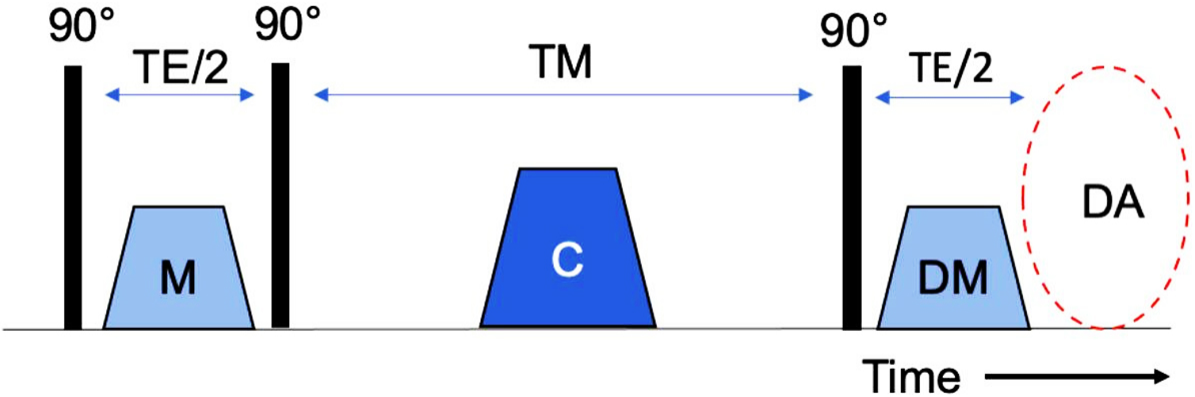
**Simplified illustration of displacement encoding with stimulated echoes (DENSE) acquisition**. After the radio frequency pulses, the modulation (M) pulse and its counter pulse (demodulation; DM) are induced. The pulses are the same size, and they are sent in the direction in which the tissue movement is to be measured. A strong pulse (crusher; C) is given between them, which removes the transversal magnetization components. The movement of the tissue can then be evaluated with the help of the difference between the modulation pulse and the counter pulse. TE/2, echo time; TM, mixing time; DA, data acquisition.

Typically, DENSE acquisition is carried out in three short axial segments of the LV: the base, the mid-ventricle and near the apex. Imaging can also be performed in the longitudinal four-chamber plane. This imaging technique is not limited to the LV but can also be used for assessing the right ventricle (RV). The DENSE technique uses a combination of radiofrequency pulses and gradients to encode myocardial displacement into the phase of the magnetic resonance imaging (MRI) signal (Fig. 1). The first pulses and the first gradient are applied at the end-diastole. After the first radio frequency pulse, a spatial magnetic field gradient pulse (modulation; M) imparts a location-dependent phase shift to the stimulated echo. A strong pulse (crusher; C) removes the transversal magnetization components. Second, another gradient pulse (demodulation; DM) removes the initial phase shift. The residual phase shift reflects tissue displacement between the two pulses [30]. Each phase image measures tissue displacement in a single direction only. For this reason, displacement requires two recordings to measure both horizontal and vertical directions [8]. This information is then further acquired and processed. The phase data is unwrapped, and spatial smoothing is applied to the displacement data to reduce noise and improve the accuracy of the displacement estimates [31]. Finally, temporal fitting is performed to generate continuous displacement fields over time, which can be used to calculate strain and other parameters of interest [31]. A recently published paper introduced an approach for calculating Lagrangian tissue displacement and strain in cine DENSE MRI. This method utilizes a regularized spatiotemporal least squares technique, providing a novel way to analyze and measure cardiac tissue movement and deformation [32].

### 2.2 Data Analysis and Dyssynchrony Parameters

**Fig. 2.**
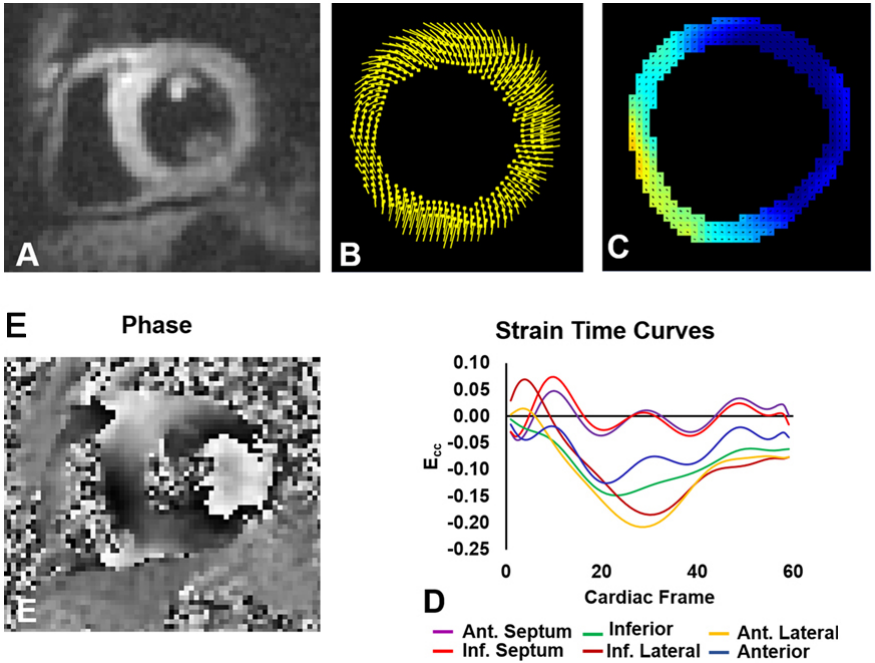
**The DENSE dyssynchrony analysis method**. Displacement encoding with stimulated echoes (DENSE): magnitude (A) and phase (E) images of a patient with heart failure and left bundle branch block. The corresponding displacement map (B), Ecc (circumferential strain) map (C), and segmental Ecc-time curve (D). Modified from Auger *et al*. 2022 [7], license: CC BY 4.0.

The DENSE dyssynchrony analysis method uses information from myocardial strains to provide insights into the changes in myocardial tissue dimension during the cardiac cycle [33]. By measuring strains, it is possible to assess the variability of contraction over time between different areas of the heart, as well as to determine the degree of synchronicity. This can be presented in milliseconds (ms) or relative to the duration of the cardiac cycle (% of RR interval [the distance between two consecutive R waves]). An example of dyssynchrony analysis is presented in Fig. 2 (Ref. [7]).

The regional heterogeneity of strains in circumferential (circumferential uniformity ratio estimate [CURE]), longitudinal (longitudinal uniformity ratio estimate [LURE]) or radial (radial uniformity ratio estimate [RURE]) planes are the most-used methods for dyssynchrony evaluation [34]. The uniformity ratio is determined from several locations in the selected plane around each imaging segment or slice and plotted versus spatial position for each time frame. The more oscillatory the plot, the greater the dyssynchrony among the segments. Plots are further subjected to Fourier analysis, and the results are averaged over space and time [35]. The obtained values are scaled between 0 (indicating dyssynchrony) and 1 (indicating synchrony) [34]. CURE with singular value decomposition (CURE-SVD) offers an advantage over standard CURE, since it does not require the selection of specific cardiac phases [36]. This is particularly important in patients with HF and dyssynchronous LV, where there is often ongoing contraction in some segments of the myocardium and simultaneous stretch in others. Yet, a previous study showed that the correlation between CURE and CURE-SVD is high (r = 0.90, *p* < 0.0001) [37]. Different DENSE dyssynchrony measurement methods are listed in Table 1 (Ref. [34, 36, 38, 39, 40, 41]).

**Table 1.** **Methods and parameters used for presenting synchronicity for displacement encoding with stimulated echoes (DENSE) in different studies**.

DENSE parameters	Explanation	Unit/Measure	Reference
CURE	Regional heterogeneity of strains in the circumferential plane	0 (dyssynchrony) – 1 (synchrony)	Bilchick *et al*. [40]
CURE-SVD	Circumferential uniformity ratio estimates with singular value decomposition	0 (dyssynchrony) – 1 (synchrony)	Ramachandran *et al*. [36]
RURE	Regional heterogeneity of strains in the radial plane	0 (dyssynchrony) – 1 (synchrony)	Budge *et al*. [34]
LURE	Regional heterogeneity of strains in the longitudinal plane	0 (dyssynchrony) – 1 (synchrony)	Budge *et al*. [34]
DI	The difference of temporal offsets of selected cardiac areas (intra or interventricular)	ms or %	Jing *et al*. [38]
			Suever *et al*. [41]
TOS	Time to onset of (circumferential) shortening — can be used to evaluate the heterogeneity of the contraction and to detect late-activating regions.	ms	Auger *et al*. [39]

CURE, circumferential uniformity ratio estimate; DI, dyssynchrony index; LURE, longitudinal uniformity ratio estimate; ms, milliseconds; RURE, radial uniformity ratio estimate; SVD, singular value decomposition; TOS, time to the onset of circumferential shortening.

The dyssynchrony index (DI) is another parameter that can be calculated by measuring the timing of contraction for each segment throughout the heart and establishing a person-specific reference strain [38]. This approach allows for a more detailed assessment of the degree of dyssynchrony in the myocardium [38]. In this method, a temporal offset (TO) is measured in ms for each segmental strain curve relative to the LV reference curve. These TOs are then mapped onto a bullseye diagram and smoothed spatially using a cubic spline. The DI is then calculated as the standard deviation of the TOs. The DI can be calculated intra-ventricularly, inter-ventricularly or separately for specific regions of the heart, such as the septum and inferolateral wall. Another parameter used in DENSE studies is the time to the onset of circumferential shortening (TOS) [39]. The TOS is the point in time when the downslope of the regional circumferential strain curve (Ecc) begins. Specifically, TOS corresponds to the moment when the rate of change of Ecc with respect to time (dEcc/dt) transitions from a value near zero to a negative value after the system detects the ECG R-wave [39]. Additionally, TOS can be utilized to assess the diversity of the contraction and identify regions that activate later. For instance, visual representations, such as bull’s-eye plots of TOS maps, can effectively illustrate synchronicity of LV contraction [39].

### 2.3 Other Methods for the Measurement of Myocardial Dyssynchrony

Myocardial MRI tagging is used to analyze the motion and deformation of the myocardium during the cardiac cycle. This technique involves the use of radiofrequency pulses to create a grid-like pattern of dark and light lines (tags) within the myocardium [1]. Harmonic phase (HARP) is a more advanced analysis technique capable of extracting and processing motion information from tagged CMR. It is obtained by calculating both motions from the horizontal direction and vertical direction, resulting in a 2D vector field [42]. Strain-encoded (SENC) MRI uses tag surfaces parallel to the image plane, combined with out-of-plane phase-encoding gradients along the slice-selected direction, to measure myocardial strain [43]. Feature-tracking (FT) analysis derives information from routine cine images by following ‘features’ across multiple images based on pattern-matching techniques without the need for additional imaging [44]. This makes the technique more efficient and less time consuming. However, the technique also has limitations, including the potential for inaccuracies due to noise and the variability in tracking performance between the different software packages [45, 46]. Moreover, FT relies on the quantification of local changes in signal intensity, which may fail when quantifying motion parallel to or inside the myocardium. Additionally, it only captures in-plane displacement, which may limit its accuracy. Compared to DENSE, FT has been shown to overestimate dyssynchrony (bias: 26 ms, *p* < 0.001, coefficient of variation [CoV]: 76%) [47].

Echocardiography is a widely used method for assessing LV volumes and ejection fraction (EF), since it displays good time and spatial resolution. Moreover, echocardiography has high availability and relatively low cost. Yet, echocardiography has some limitations, including its relatively high inter-observer variability and, in some cases, poor visibility, particularly on the right side of the heart [19]. However, the consistency of echocardiographic parameters used to measure dyssynchrony has been modest, according to the Predictors of Response to CRT Trial (PROSPECT), which is one of the largest and most widely referenced investigations in this field [19].

Dyssynchrony assessment with single-photon emission computerized tomography (SPECT) is based on regional changes in LV counts during the cardiac cycle. Phase analysis quantifies the heterogeneity of contraction onset times in various regions of the LV and displays the results as a histogram. The wider the histogram, the more substantial the dyssynchrony. Dyssynchrony can also be measured using other nuclear imaging techniques such as multi-gated acquisition radionuclide angiography and gated blood-pool SPECT [48]. However, there are several disadvantages to this method, including radiation and relatively low spatial resolution. Also, RV or atrial analysis is not possible with this technique.

RV function is an important indicator of various cardiopulmonary diseases [49]. However, the complex anatomy and thin wall of the RV make it challenging to assess its function using conventional imaging techniques [50]. Echocardiography is widely used for assessing RV function but has limitations due to the retrosternal position of the RV. Three-dimensional echocardiography and myocardial deformation imaging may help overcome these limitations. Nuclear imaging techniques can estimate LV dyssynchrony but are unsuitable for assessing RV function. MRI offers excellent tissue contrast and multi-slice imaging capabilities, making it useful for assessing RV mechanics. Yet, only a few MRI studies have investigated RV synchronicity and contraction patterns.

### 2.4 Clinical Applications for Dyssynchrony Measurements

CRT is a treatment for advanced HF that involves implanting a device to resynchronize LV contractions. However, nearly a of third patient selected utilizing the traditional inclusion criteria for CRT (EF ≤35%, QRS duration ≥120 ms, left bundle branch block, with moderate or severe HF symptoms) have an inadequate response [18, 19]. It has been shown that dyssynchrony measurement can help in CRT patient selection [51, 52, 53, 54] and can also be useful for monitoring disease progression and response to treatment in patients with HF [55]. Moreover, measuring LV dyssynchrony can enhance our understanding of the underlying pathophysiology of HF [56].

## 3. Method For the Systematic Review

### 3.1 Search Criteria

Four electronic databases were searched: PubMed (US National Library of Medicine, http://www.ncbi.nlm.nih.gov/pubmed), Scopus (Elsevier, http://www.scopus.com/), Web of Science (Thomson Reuters, http://apps.webofknowledge.com/) and Cochrane (https://www.cochrane.org/). These databases are widely recognized in the scientific community as reliable sources of scholarly publications and were chosen for their comprehensive coverage of medical research literature. By utilizing multiple databases, this study aimed to ensure a thorough and comprehensive search for relevant papers.

To identify relevant studies, the following search criteria were used: (DENSE OR ‘displacement encoding with stimulated echoes’ OR CURE) AND (dyssynchrony* OR asynchron* OR synchron*) AND (MRI OR ‘magnetic resonance’ OR CMR). The search was conducted for articles published from January 2000 through January 2023 and included the use of relevant keywords and their variations.

### 3.2 Data Analysis

First, the researchers excluded duplications. Next, they analyzed the titles and abstracts of various studies and only included those that reported results considering CMR related imaging. The studies had to meet specific inclusion criteria, such as being clinical research or technical development studies and having been published in English. The researchers excluded reviews, general overview articles and case reports. The final articles were those that considered the DENSE technique and especially dyssynchrony imaging. During the selection process, two readers worked together to decide which articles to include in the analysis. The researchers extracted information, such as authors, year of publication, sample size and diagnostic modality, from each study.

## 4. Results

**Fig. 3.**
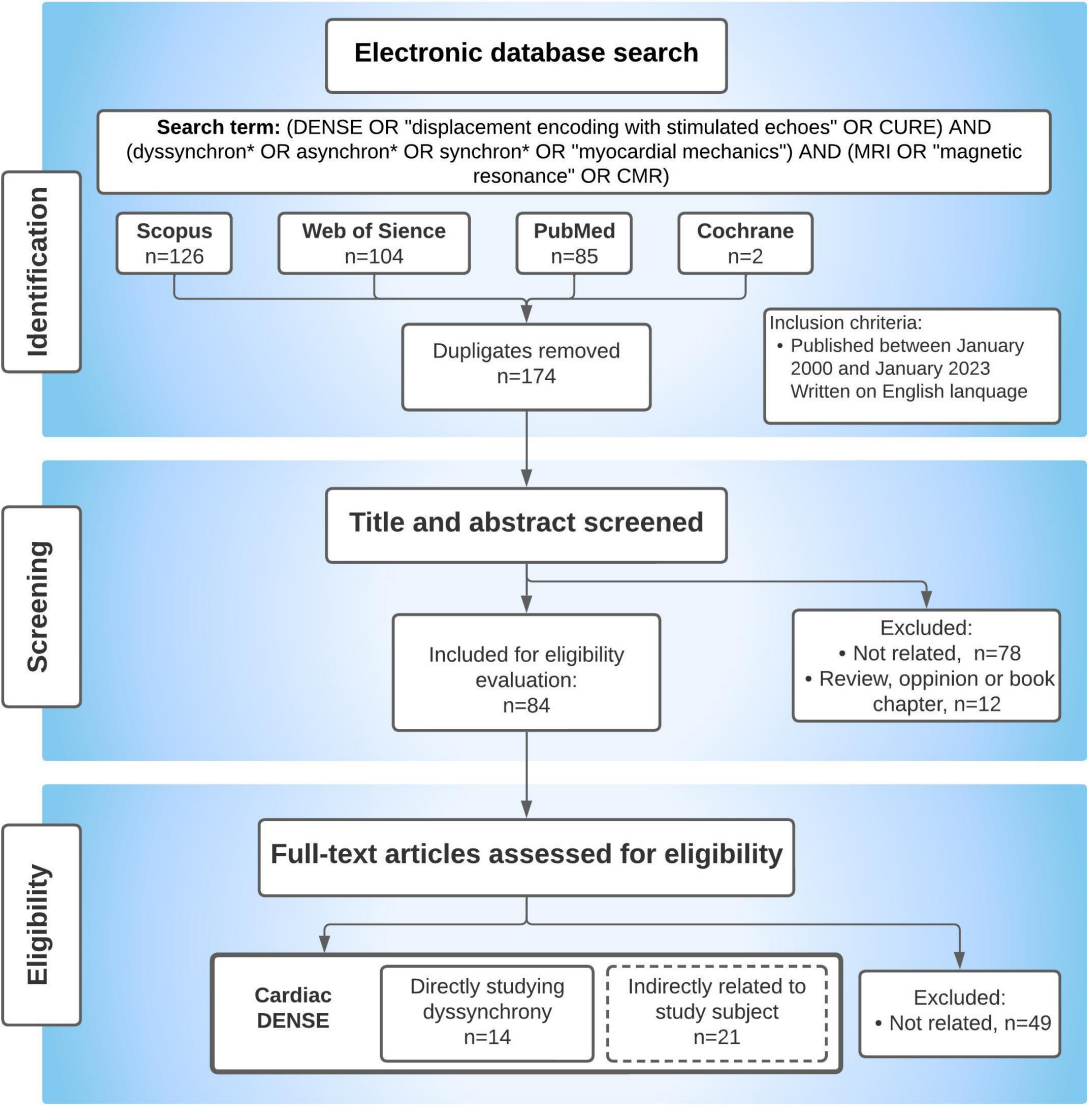
**Illustration of the review search process through the Scopus, Web of Science, Pubmed, and Cocharine databases**. The process involved first removing duplicates and next screening titles, abstracts and full articles to identify relevant papers. Ultimately, 14 papers that examined cardiac dyssynchrony using displacement encoding with stimulated echoes (DENSE) were included in the final analysis. Additionally, other DENSE research papers that were indirectly related to dyssynchrony were also considered as background material and will be discussed in the paper where applicable. This figure serves to visually explain the steps taken to identify the papers included in this study and provides context for the analysis. CURE, circumferential uniformity ratio estimate; MRI, magnetic resonance imaging; CMR, cardiovascular magnetic resonance.

With the search terms used in this systematic review, 126 articles were found from Scopus, 104 articles from Web of Science, 85 articles from PubMed and 2 articles from Cochrane (Fig. 3). After duplicates were removed 174 papers remained. First, titles and abstracts were screened. Seventy-eight papers were not related to the subject and were excluded for that reason. Twelve papers were excluded because they were either review articles, book chapters or opinions. Next, the remaining 84 full-text articles were read. Forty-nine articles were excluded because they were not focused on DENSE. Fourteen papers directly studied dyssynchrony estimation with DENSE (Tables 2A,2B, Ref. [34, 36, 37, 39, 40, 41, 47, 57, 58, 59, 60, 61, 62, 63]) and were discussed in more detail in this review. Studies about DENSE without measurement of dyssynchrony were used as background material (n = 21, Table 3, Ref. [4, 5, 6, 8, 12, 13, 14, 15, 16, 17, 21, 22, 23, 24, 25, 26, 27, 64, 65, 66, 67]). Out of the fourteen DENSE-based dyssynchrony studies, nine studies were conducted only on humans [37, 39, 40, 41, 47, 58, 59, 60, 62], while four studies were conducted only on animals (mice or canine) [34, 57, 61, 63]. One study combined results from both human and animal studies [36]. Seven of the human studies and two of the animal studies were related to HF or CRT [28, 36, 37, 39, 40, 59, 60, 62, 63]. One of the human studies focused on the relationship between dyssynchrony and fibrosis in a repaired Tetralogy of Fallot (rTOF) patient population [60], while another study examined obesity’s influence on DENSE in mice [61]. Two studies compared DENSE results with other CMR methods of evaluating dyssynchrony, such as tissue tagging and feature tracking [34, 47]. One paper provided 3D DENSE values for healthy human controls [41], and two papers provided information on the reducibility of dyssynchrony parameters obtained with DENSE [41, 57]. The sample sizes of the studies ranged from 9 to 200. The studies used various parameters to estimate dyssynchrony, including CURE, CURE-SVD, LURE, RURE, DI, TOS, ms and time/percentage difference on contraction onset. Some studies used several parameters to measure dyssynchrony.

**Table 2A.** **Published human research utilizing displacement encoding with stimulated echoes (DENSE) to estimate cardiac dyssynchrony**.

Year of publ.	Topic	Number of subjects			Dyssynchrony measurements					MRI devise	Reference
		Total	Healthy	Disease	Parameter	LV	RV	2D	3D	(Tesla)	
2022	CRT response (sex influence)	200		HF (200)	CURE-SVD	x		x		Unknown	Bivona *et al*. [58]
2022	CRT response (ML)	200		HF (200)	CURE-SVD	x		x		Unknown	Bivona *et al*. [59]
2021	CRT response	50		HF (50)	CURE-SVD	x		x		Siemens Aera (1.5)	Gao *et al*. [62]
2020	Arrhythmia risk after CRT	100		HF (100)	CURE-SVD	x		x		Siemens Aera/Avanto (1.5)	Bilchick *et al*. [37]
2018	Comparison FT and DENSE	88	na	Different	ms	x		x		Siemens Avanto (1.5)/Trio (3)	Wehner *et al*. [47]
2017	Normal DENSE values and reproducibility	50	50		DI	x	x		x	Siemens Trio (3)	Suever *et al*. [41]
2017	CRT lead placement	56	6	HF (50)	TOS	x		x		Siemens Avanto (1.5)	Auger *et al*. [39]
2017	LV fibrosis and mechanics	40		rTOF (40)	DI	x		x		Siemens Avanto (1.5)	Haggerty *et al*. [60]
2015	Selection of CRT candidates	80		HF (80)	CURE, CURE-SVD	x		x		Siemens Avanto (1.5)	Ramachandran *et al*. [36]
2014	CRT response	75		HF (75)	CURE	x		x		Siemens Avanto (1.5)	Bilchick *et al*. [40]

Footnote: 2D, two-dimensional; 3D, three-dimensional; CRT, cardiac resynchronization therapy; CURE, circumferential uniformity ratio estimate; CURE-SVD, circumferential uniformity ratio estimates with singular value decomposition; DI, dyssynchrony index; FT, feature tracking; HF, heart failure; LV, left ventricle; ML, machine learning; MRI, magnetic resonance imaging; na, information not available; publ., publication; rTOF, repaired Tetralogy of Fallot; RV, right ventricle; TOS, time to the onset of circumferential shortening.

**Table 2B.** **Published animal research utilizing displacement encoding with stimulated echoes (DENSE) to estimate cardiac dyssynchrony**.

Year of publ.	Topic	Animal study (n)			Dyssynchrony measurements			MRI devise	Reference
		Species (n)	Healthy	Disease	Parameter	LV	2D	(Tesla)	
2015	Selection of CRT candidates	Canine (13)	3	HF (10)	CURE, CURE-SVD	x	x	Siemens Avanto (1.5)	Ramachandran *et al*. [36]
2013	Obesity influence on DENSE	Mice (10)	5	5	CURE, RURE	x	x	Bruker ClinScan (7)	Kramer *et al*. [61]
2013	Reducibility of DENSE	Mice (9)	5	4	CURE, RURE	x	x	Bruker ClinScan (7)	Haggerty *et al*. [57]
2012	Comparison of DENSE and tissue tagging	Canine (13)	3	HF (10)	CURE, LURE, RURE, ms	x	x	Siemens Avanto (1.5)	Budge *et al*. [34]
2010	Assessment of dyssynchrony with DENSE*	Canine (10)		HF (10)	CURE, RURE, ms	x	x	Siemens Avanto (1.5)	Bilchick *et al*. [63]

Footnote: 2D, two-dimensional; CRT, cardiac resynchronization therapy; CURE, circumferential uniformity ratio estimate; CURE-SVD, circumferential uniformity ratio estimates with singular value decomposition; DI, dyssynchrony index; HF, heart failure; LURE, longitudinal uniformity ratio estimate; LV, left ventricle; MRI, magnetic resonance imaging; n, number of study animals which of the dyssynchrony was evaluated; publ., publication; RURE, radial uniformity ratio estimate. *Conference abstract.

**Table 3.** **Published research on displacement encoding with stimulated echoes (DENSE) excluding dyssynchrony studies**.

Year of publ.	Topic	Human (n)	Animal (n)	Phant.	DENSE measurement							MRI devise	Reference
					Strain	Torsion	Twist	2D	3D	LV	RV	(Tesla)	
2021	3D displacement tracking and R	7			x	x			x	x	x	Siemens Aera (1.5T)	Carruth *et al*. [13]
2019	Drug influence on heart		Rat (9)		x	x			x	x		Bruker ClinScan (7T)	Zang X al. [64]
2017	Reproducibility	17			x	x	x	x		x		Siemens Aera (1.5T)	Lin *et al*. [6]
2017	Reproducibility		Rat (10)	x	x	x			x	x		Bruker ClinScan (7T)	Zhang *et al*. [25]
2016	Fast imaging method	14			x			x		x		Siemens Avanto (1.5T)	Chen *et al*. [4]
2016	Multiparametric strain Z-score	36			x				x	x		Siemens Avanto (1.5T)	Kar *et al*. [16]
2015	Comparison 3T vs 1.5T and R	16			x	x		x		x		Siemens Aera (1.5T) or Trio (3T)	Wehner *et al*. [65]
2015	Encoding technique and R	20			x		x	x		x		Siemens Trio (3T)	Wehner *et al*. [5]
2015	Influence on obesity on DENSE		Mice (30)		x	x		x		x		Bruker ClinScan (7T)	Haggerty *et al*. [26]
2014	Reproducibility	62	Mice (135)	x	x	x		x		x		Bruker ClinScan (7T), Siemens Trio (3T) or Avanto (1.5T)	Suever *et al*. [66]
2014	3D technique		Rat (1)		x	x	x		x	x		Bruker Biospec (7T)	Gomez *et al*. [14]
2014	Surface fitting algorithm		Mice (17)	x				x		x		Bruker Biospec (7T)	Haggerty *et al*. [21]
2013	Mouse and human mechanical phantom				x	x		x		x	x	Siemens Espree (1.5T)/General Electrics (7T)	Zhong *et al*. [22]
2012	Technical	10			x			x		x		Siemens Avanto (1.5T)	Gilliam & Epstein [8]
2012	Right ventricular mechanics	5		x	x				x	x	x	Siemens Avanto (1.5T)	Auger *et al*. [12]
2012	Evolution 3D strains 2D images	1			x			x		x		Philips Ahieva (3T)	Kindberg *et al*. [17]
2011	Mice heart assessment in 3D		Mice (7)		x	x	x		x	x		Brucer ClinScan (7T)	Zhong *et al*. [15]
2010	Healthy/comparison	5		x	x	x	x	x	x	x		Siemens Avanto (1.5T)	Zhong *et al*. [23]
2009	Mathematical model		Sheep (7)		x				x	x		Siemens Sonata (1.5T)	Liu *et al*. [24]
2007	Novel method DENSE		Mice (14)		x			x		x		Unknown	Gillian *et al*. [27]
2005	Measuring cardiac mechanics		Mice (7)		x	x	x	x		x		Varian INOVA (4.7T)	Gilson *et al*. [67]

Footnote: Publ., publication; n, number of study subjects/animals which of the dyssynchrony was evaluated (other parameters might have been studied in bigger population); Phant., mechanical or mathematical phantom; MRI, magnetic resonance imaging; 2D, two-dimensional; 3D, three dimensional; LV, left ventricle; RV, right ventricle; R, reproducibility.

Six of the human studies were conducted using 1.5T MRI machines, specifically Siemens Aera or Avanto. One study combined results from 1.5T Siemens Avanto [36, 37, 39, 40, 60, 62] and 3T Siemens Trio [47], while one human study used the 3T Siemens Trio machine [41]. There was no information available on the scanning device used in the two studies provided by the same research group (Table 2A). 7T Brucer ClinScan was used in two mice studies. Canine studies were performed in Siemens Avanto 1.5T machines (Table 2B). Many of the studies reported their scanning procedures, although not all of them did [58, 59, 63]. The temporal resolution reported was typically 17 ms, although some studies reported lower temporal resolutions [41, 47, 60]. The slice thickness was commonly 8 mm, although in mice studies, it was as low as 1 mm. The pixel size was most commonly 2.8 × 2.8 ${\mathrm{mm}}^{2}$, although some studies used smaller pixel sizes. The image matrix was reported in two studies as either 128 × 128 or 180 × 180. The field of view (FOV) typically ranged from 250 × 250 to 360 × 360 ${\mathrm{mm}}^{2}$, while one study reported an FOV of 340–400 ${\mathrm{mm}}^{2}$ [36]. The reported flip angle was either 15° or 20°. The displacement encoding frequency ke (cycles/mm) ranged from 0.04 to 1.0. The repetition time was stated to be either 7.1 or 17 ms, and the echo time ranged from 0.67 to 1.9.

### 4.1 Normal Values and Reproducibility of DENSE Dyssynchrony Analysis

We identified only one study that provided DENSE values for a healthy human population. The study from Suever *et al*. [41], involved 50 healthy individuals and measured global cardiac mechanics for both LV and RV using a 3 Tesla (Tim Trio) machine. The timing of contraction was assessed across the ventricles by calculating the mechanical activation delay in ms for each segment in relation to a patient-specific reference curve and further converted from ms to percent of the cardiac cycle delay time to provide DI. The mean global 3D DENSE values for LV were 25.0 ± 6.9 ms and DI 3.4 ± 1.0%. For RV, the mean global 3D DENSE values were 23.3 ± 8.3 ms and DI 3.1 ± 1.1%. Inter-ventricular values were –0.7 ± 10.6 ms and DI –0.0 ±1.5% respectively.

Previous studies have demonstrated that the reproducibility of DENSE strain is superior to that of tagging [6]. The reproducibility of DENSE dyssynchrony parameters has been evaluated in several studies. Suever *et al*. [41] also studied the inter-observer variability of DI from 10 healthy individuals and the inter-study reproducibility from 6 healthy individuals using the CoV. For LV DI, the CoV was 6%, and for RV DI, the CoV was 10%. The inter-study reproducibility for LV DI was 12% and for RV DI 11%. Inter-observer variability was also reported for DI in the rTOF population. For the LV, the CoV was 9% and the intra-class correlation (ICC) 0.76 (95% CI 0.32–0.93); for RV DI, the CoV was also 9%, and the ICC was 0.84 (95% CI 0.51–0.96); and for inter-ventricular DI, the CoV was 7% and the ICC 0.90 (95% CI 0.66–0.97) [38]. Haggerty *et al*. [57] studied reproducibility on five healthy mice (C57BL/6) and four mice with impaired cardiac function (diet-induced obesity), which were imaged with a 7T ClinScan MR system. CURE and RURE were used as indices of synchrony. The inter-observer variability, expressed as (CoV), was 1% for CURE and 3% for RURE. Similarly, the inter-study variability was 1% for CURE and 3% for RURE.

### 4.2 DENSE and Clinical Response for CRT

We found seven studies on DENSE dyssynchrony analysis related to CRT response. However, only one provided optimal cut-off values with accuracy analysis predicting patient outcome. Bilchick *et al*. [40] showed that patients who have CURE below 0.70, a delay in electrical contraction at the LV lead position and no scarring in that location are more likely to experience the benefits of CRT. The study consisted of 75 patients and 40 (53%) patients identified with positive CRT response. Multivariable logistic modeling accurately identified CRT responders (area under the curve: 0.95 [*p* < 0.0001]) based on CURE (odds ratio [OR]: 2.59/0.1 decrease), delayed circumferential contraction onset at left ventricular lead position (LVLP) (OR: 6.55), absent LVLP scar (OR: 14.9), and the time from QRS onset to LVLP electrogram (OR: 1.31/10 ms increase). Patients with a favorable CMR profile, characterized by a CURE less than 0.70, no LVLP scar, and delayed electrical conduction onset at LVLP, demonstrate better overall survival compared to patients without dyssynchrony as determined by a CURE score of 0.70 or higher [40]. In that study optimal cut-off value was for CURE <0.70 with sensitivity or 100% and specificity of 65.7%. Ramachandran *et al*. [36] showed that patients with positive CRT response had statistically more LV dyssynchrony and lower CURE (0.38 *vs*. 0.75, *p* < 0.0001) and CURE-SVD (0.45 *vs*. 0.84, *p* < 0.0001) compared to non-responders. Another study compared DENSE to other CMR dyssynchrony methods and showed that CURE-SVD measured with DENSE had a stronger correlation with CRT response (r = –0.57, *p* < 0.0001) than CURE-SVD with FT (r = –0.28, *p* = 0.004) [37].

In a recently published prospective study of 200 patients, the influence of sex on CRT response was evaluated. The study found that females had more mechanical dyssynchrony before CRT installation compared to males, as measured by the CURE-SVD parameter (0.52 *vs*. 0.61, *p* = 0.04) [58]. Additionally, female patients had a smaller LV end-diastolic volume index and a lower frequency of both late gadolinium enhancement and ischemic cardiomyopathy. To further analyze the data, patients were categorized into four groups based on sex and cardiomyopathy type (ischemic or non-ischemic). Female patients with non-ischemic cardiomyopathy had the lowest pre-CRT CURE-SVD, indicating more dyssynchrony (*p* = 0.003), but also had the most favorable response for LV function. Moreover, female patients had better three-year survival compared to men. Another study conducted by the same research group and utilizing the same dataset delved into the application of machine learning (ML) for predicting CRT response and long-term survival [59]. The study examined the associations of 39 baseline features, and ML-generated response clusters were evaluated, with cross-validation assessing the associations of clusters with four-year survival. The study found that lower CURE-SVD values (indicative of more dyssynchrony) were associated with greater CRT response. Additionally, the top three pre-CRT predictors were CURE-SVD, pre-CRT B-type natriuretic peptide and pre-CRT peak ${\mathrm{VO}}_{2}$. The resulting model was able to provide reassurance to approximately 62% of patients that their four-year survival was expected to be favorable, while also identifying 16% of patients who would benefit from further evaluation or advanced HF therapies after CRT.

Bilchick *et al*. [37] studied the prognostic value of DENSE for CRT patients by combining the Seattle Heart Failure Model (SHFM) and DENSE CURE-SVD circumferential strain dyssynchrony parameter. The SHFM score provides estimates for one-, two- and three-year survival with the use of the clinical, pharmacological, device and laboratory characteristics [68]. In a cohort of 100 patients who were followed a median of 5.3 years, CURE-SVD and SHFM were independently associated with the primary endpoint of death, heart transplantation, LV assist device and appropriate implantable cardioverter-defibrillator therapies (SHFM: hazard ratio (HR) 1.47/standard deviation (SD), 95% CI 1.06–2.03, *p* = 0.02; CURE-SVD: HR 1.54/SD, 95% CI 1.12–2.11, *p* = 0.009) [37].

Previous studies have suggested that using cardiac imaging could be advantageous in guiding the placement of the CRT lead [69]. Auger *et al*. [39] used TOS to evaluate the synchronicity and the latest contraction site of the LV. They showed that TOS was associated with an improvement in LV reverse remodeling with CRT.

### 4.3 Measuring the right Ventricle with DENSE

Suever *et al*. [41] determined RV geometry from 50 healthy individuals (age: 26 ± 8 years, 46% male) with no history of cardiovascular disease by using DENSE. The timing of contraction was assessed across the entire RV by calculating the mechanical activation delay in ms for each segment in relation to a patient-specific reference curve and further converted from ms to percent of the cardiac cycle delay time to provide DI. For the RV, the mean 3D DENSE values were 23.3 ± 8.3 ms and DI 3.1 ± 1.1.

### 4.4 DENSE Dyssynchrony Studies on Fibrosis and Obesity

Haggerty *et al*. [60] studied 40 patients with rTOF. They found a significant association between LV dyssynchrony and myocardial fibrosis in their study of rTOF patients using spiral cine DENSE and modified Look-Locker inversion recovery (MOLLI) T1-mapping. Specifically, they found that extracellular volume fraction, a measure of myocardial fibrosis, was positively associated with a log-adjusted DI (β = 0.67), suggesting a correlation between cardiac fibrosis and mechanical dyssynchrony.

Kramer *et al*. [61] randomized ten 12-week-old C57BL/6 J mice to a high-fat (60% of calories from fat) or low-fat (10% of calories from fat) diet. After five months on the diet, mice were imaged with a 7 T Bruker ClinScan using a cine DENSE protocol. To evaluate the myocardial contraction, LV strains were calculated and used to quantify LV synchrony using CURE and RURE indices [61]. Kramer *et al*. [61] found that obese mice had a 15% increase in LV mass compared to the control mice. Interestingly, there was no difference in EF between the groups (*p* = 0.056). The synchrony of contraction in the LV was decreased in obese mice; RURE was 0.95 ± 0.02 in the control mice and 0.91 ± 0.03 in the obese mice (*p* = 0.032). CURE did not differ across the two groups of mice (*p* = 0.151).

## 5. Discussion

DENSE is a promising modality for quantifying myocardial dyssynchrony, particularly prior to the CRT implantation, since it can help in identifying responders to CRT, predict long-term survival and may detect optimal pacing sites. Combining imaging and clinical parameters can enhance the identification of the best candidates for CRT and lead to improved patient outcomes. Moreover, DENSE has advantages over other MRI-based dyssynchrony estimation methods, such as improved spatial and temporal resolutions. However, it requires a special imaging sequence.

DENSE has advantages over other dyssynchrony estimation methods. Each phase pixel occupied by tissue is proportional to a displacement value indicating the location of the occupying tissue element when the DENSE encoding pulses are applied [8]. For this reason, DENSE has contributed to improved spatial resolution in assessing myocardial deformation compared to previous methods such as tissue tagging may theoretically help identify subtle changes in myocardial function that might not be detected by conventional MRI imaging techniques. DENSE also has good temporal resolution (~17 ms at a heart rate of 60 bpm) [45]. This enables clinicians to obtain more detailed information about cardiac function and dyssynchrony, which can help guide decisions regarding treatment options. Additionally, novel techniques have been developed to increase the signal quality of DENSE imaging [5, 70]. Moreover, acquisition times have shortened, and scan time is about 12–14 heartbeats per imaging plane [3]. The main limitation of DENSE is that it requires a special imaging sequence to be acquired, and the post-processing analysis can be time consuming. This is particularly important in clinical settings where time is of the essence, and faster imaging protocols can help reduce patient discomfort and improve workflow efficiency.

The findings from recent studies on DENSE imaging in CRT patients are promising. These studies suggest that DENSE could be a valuable tool for quantifying myocardial dyssynchrony and detecting CRT responders. CRT responders tend to have more dyssynchrony than non-responders, and DENSE been shown to be more reproduceable and more effective at identifying this dyssynchrony than other modalities, such as FT or tagging. In addition, the studies have highlighted the importance of combining DENSE values with clinical background information to enhance the predictive value of the study. This approach can help clinicians identify the best candidates for CRT, predict long-term survival and detect optimal pacing sites. For instance, Bilchick *et al*. [40] showed that patients with dyssynchrony and myocardium without scar in the area of the CRT lead were more likely to benefit from CRT. This finding is in line with previous studies performed with other methods [71, 72]. Moreover, TOS was shown to associate with an improvement in LV reverse remodeling with CRT [39]. This hints that TOS could theoretically be used as a tool to achieve optimal LV lead placement in CRT, but further clinical studies are needed. Interestingly, there appears to be a sex difference in the results of CRT, with females with non-ischemic cardiomyopathy exhibiting more mechanical dyssynchrony before CRT implantation compared to males, but also having a more favorable response for LV function and better prognosis [58]. These findings highlight the potential importance of taking gender into account when considering patients for CRT. Despite the promising results of mechanical dyssynchrony estimation, current major guidelines for patient selection do not include any criteria based on imaging for patients with a widened QRS complex. This suggests a critical area for future research, where cutting-edge imaging techniques like CMR could potentially improve patient selection for CRT. It is also noteworthy that CMR can provide other useful information for guiding the optimal placement of the CRT lead, such as information of the venous anatomy.

Multimodality cardiovascular imaging plays a central role in the diagnosis and follow-up of patients with congenital heart disease. Clinicians and scientists are interested not only in cardiac morphology but also in the maladaptive ventricular responses that render this population predispose to adverse outcomes. For this reason, there is rising interest in using DENSE dyssynchrony measurement in this patient group. Other applications for DENSE might include drug studies, gene therapy studies and medical device research. The acute changes in patients on new therapies can be subtle. For this reason, DENSE might be useful in detecting early changes in the therapy before other conventional measurements can show them. DENSE could also give new information about the functionality and efficacy of current vector and gene delivery systems. This also applies to understanding the involvement of disease cascade in different cardiovascular diseases. DENSE could potentially provide novel information on the pathophysiology behind specific cardiovascular diseases and help to find particular cardiac phenotypes that are likely to be more responsive to different therapies. Further, DENSE can be safely used for long-term follow-up since it lacks radiation burden. Morever, it has very low inter-observer variability.

DENSE is a promising imaging technique that can provide valuable information about myocardial function and dyssynchrony. However, there are some challenges that need to be addressed in order to facilitate its wider adoption in clinical practice. One of the main challenges with DENSE is the time-consuming nature of the CMR scanning and image processing required. Although there are tools available to speed up the process, the additional image sequence required for DENSE analysis can make it a time-intensive process. Beyond this, DENSE analysis requires an additional image sequence recorded beyond conventional clinical CMR imaging settings. Also, motion artifacts and partial volume effects can affect the accuracy of DENSE analysis, adding to the challenges of using this technique. While there are challenges involved with DENSE, the potential benefits of DENSE in providing novel information on the pathophysiology of specific cardiovascular diseases make it a valuable method in cardiovascular research. Yet, further research is necessary to advance the DENSE technique. Particularly in establishing more robust normal values obtained from larger, diverse, healthy populations, validating its diagnostic and prognostic value in comparison to other imaging techniques for various cardiovascular diseases, and developing automated and standardized analysis methods to enhance its clinical applicability and seamless integration into routine practice, including cost effectiveness analyses. As CMR technology continues to evolve and improve, it will be interesting to see how DENSE and other imaging modalities will be incorporated into clinical practice and how they can be used in conjunction with other imaging techniques to provide a more comprehensive understanding of cardiac function and heart diseases. Assessment of the RV is a challenging task due to its complex anatomy and location in the chest. DENSE can potentially overcome this challenge and provide information on RV synchronicity. Suever *et al*. [41] RV study lays a foundation for future research to explore deviations from healthy contraction patterns, potentially leading to new insights into the development and prognosis of various RV right ventricle diseases. Over the last few decades, CMR imaging has emerged as an indispensable tool, allowing non-invasive and simultaneous assessment of whole-heart morphology and function. Artificial intelligence (AI) is known to excel at segmentation and pattern recognition. These solutions might help in making the analysis process faster and more feasible for clinicians. Also, AI might present new opportunities to detect early signs of diseases that have not yet been diagnosed.

## 6. Conclusions

In conclusion, DENSE is a promising modality for the quantification of myocardial function and dyssynchrony. It has advantages over other CMR methods in evaluating the displacement of the LV wall, and its high reproducibility is an undeniable benefit. DENSE dyssynchrony analysis might help in detecting those patients are responding to CRT, finding the best setting for the CRT device and even providing information on RV synchronicity. DENSE could also give new information on the pathophysiology behind HF. However, there are some challenges related to dyssynchrony measurement with DENSE, and its use in everyday clinical practice is not yet feasible. In the future, it may become possible to obtain DENSE quickly and with automated analyses, making it a realistic method for everyday use. Additionally, advancements in CMR technology and the integration of AI solutions could potentially address existing challenges and result in the widespread adoption of DENSE in clinical practice.

## Supplementary Material

Supplementary material associated with this article can be found, in the online version, at https://doi.org/10.31083/j.rcm2409261.
